# FAPI Tracer *en Vogue*: Evaluating [^68^Ga]Ga-DATA^5m^.SA.FAPi for Molecular Imaging of Pulmonary Fibrosis

**DOI:** 10.3390/ph19010034

**Published:** 2025-12-23

**Authors:** Victoria Weissenböck, Michaela Schlederer, Latifa Bakiri, Johanna Schaffenrath, Erwin F. Wagner, Frank Rösch, Marcus Hacker, Lukas Kenner, Cécile Philippe

**Affiliations:** 1 Department of Biomedical Imaging and Image-Guided Therapy, Division of Nuclear Medicine, Medical University of Vienna, 1090 Vienna, Austria; 2 Department of Pathology, Medical University of Vienna, 1090 Vienna, Austria; 3 Department of Laboratory Medicine, Medical University of Vienna, 1090 Vienna, Austria; 4 Genes and Disease Laboratory, Department of Laboratory Medicine, Medical University of Vienna, 1090 Vienna, Austria; 5 Genes and Disease Laboratory, Department of Dermatology, Medical University of Vienna, 1090 Vienna, Austria; 6 Institute of Nuclear Chemistry, Johannes Gutenberg University Mainz, 55128 Mainz, Germany; 7 Ludwig Boltzmann Platform for Comparative Laboratory Animal Pathology, 1090 Vienna, Austria; 8 Unit of Laboratory Animal Pathology, University of Veterinary Medicine, 1210 Vienna, Austria

**Keywords:** FAP, fibrosis, PET, bleomycin, *fra*-2

## Abstract

**Background/Objectives**: Radiolabeled fibroblast activation protein inhibitors (FAPIs) are emerging as promising imaging agents assessing fibrotic diseases. This study evaluates [^68^Ga]Ga-DATA^5m^.SA.FAPi for imaging pulmonary fibrosis in two mouse models, bleomycin-induced (BLM) and a transgenic (*fra*-2^tg^) model, both displaying characteristics of human pulmonary fibrotic diseases. **Methods**: In the BLM model, C57BL/6 mice were treated with bleomycin or isotonic sodium chloride (controls) for 4, 5, and 6 weeks, followed by [^68^Ga]Ga-DATA^5m^.SA.FAPi PET/CT scans. *Fra*-2^tg^ mice and wildtype (WT) littermates underwent at 7, 11, and 18/19 weeks of age a PET/CT scan. The selected timepoints correspond to early, middle, and late disease stages for each model. Imaging was complemented by ex vivo quantification, histological, and immunohistochemical (IHC) analyses. **Results**: In BLM mice, pulmonary [^68^Ga]Ga-DATA^5m^.SA.FAPi uptake showed a trend toward increase as early as 5 weeks of treatment compared with the controls, which was confirmed by ex vivo analysis (BLM: 3.31 ± 0.29%ID/g, n = 5; control: 1.61 ± 0.29%ID/g, n = 4; *p* = 0.0035). In *fra*-2^tg^ mice, no significant differences could be detected. IHC revealed elevated pulmonary FAP expression specifically at early (BLM) and mild (*fra*-2^tg^) disease stages, whereas for BLM, tracer uptake was more pronounced at later stages. **Conclusions**: Our findings complement and extend observations from previous studies and support the potential of FAPI tracers as molecular imaging agents for pulmonary fibrosis.

## 1. Introduction

Radiolabeled fibroblast activation protein inhibitors (FAPIs) have gained significant attention in recent years as novel agents in tumor imaging, demonstrating efficacy comparable to that of standard [^18^F]fluorodeoxyglucose ([^18^F]FDG) positron emission tomography/computed tomography (PET/CT). Remarkably, the application of FAPI tracers is expanding beyond oncology, with a growing interest in their potential for imaging fibrotic processes. An increasing number of publications are exploring these alternative purposes, underscoring the versatility of FAPI tracers as a promising tool for assessing fibrotic tissue [1,2,3,4,5,6,7,8,9,10,11,12,13,14,15].

In 2022, over 100 million individuals were impacted by organ fibrosis, with 4.6 million suffering from pulmonary fibrotic diseases [16,17]. The pathogenesis is marked by the activation of fibroblasts and excessive deposition of connective tissue, specifically collagen. This pathological process leads to the disruption of organ function, potentially culminating in complete organ failure [18,19]. Consequently, fibrotic diseases are responsible for an estimated 45% of deaths in industrialized nations, with lung and cardiac fibrosis being the predominant contributors [20]. This substantial prevalence highlights the urgent need for enhanced diagnostic and effective therapeutic strategies for these serious and life-threatening conditions.

The most commonly used preclinical model for studying pulmonary fibrosis is the bleomycin (BLM)-induced mouse model [21,22,23,24]. Although BLM, an anti-neoplastic agent, can be used to treat certain tumors, its pulmonary toxicity has been recognized since the earliest clinical studies, where 42% of the patients developed lung damage [25,26]. Paradoxically, this deleterious side effect provided a new important application in preclinical research. The BLM-induced mouse model is one of the oldest and best-known animal models for mimicking human pulmonary fibrotic diseases [22,27,28,29,30,31,32,33,34,35,36,37,38,39,40,41,42,43,44,45]. The conventional method of administration is intratracheal, followed by intranasal, intravenous, and intraperitoneal [22,27,28,32,35,38,40,46]. Regardless of the administration route, BLM induces parenchymal lung inflammation, characterized by epithelial cell injury with reactive hyperplasia, along with the activation and differentiation of fibroblasts into myofibroblasts [44]—a hallmark of fibrotic diseases and the target of FAPI tracers. In our study, BLM was administered intraperitoneally to induce systemic fibrosis and to align this model with our second mouse model, transgenic Fos-related antigen 2 (*fra*-2^tg^) mice, which is characterized by systemic fibrosis. In *fra*-2^tg^ mice, collagen deposition is most pronounced in the lungs but can also occur in other organs, including heart, liver, and thymus [47].

Extensive research on individual Fos proteins using both gain- and loss-of function mouse models has revealed their crucial roles in the proliferation and differentiation of bone cells [48,49,50]. Although the transcription factor AP-1, which consists of Jun and Fos proteins, is known to regulate various stress responses such as proliferation, apoptosis, and inflammation, direct genetic evidence linking Fra-2 or other Fos proteins specifically to fibroproliferative disease mechanisms is limited [47,51]. To address this gap, Eferl et al. generated the *fra*-2^tg^ mouse model in 2008 [47]. Their study reported that Fra-2 has profibrogenic activity, as its ectopic expression in transgenic mice led to fibrosis primarily in the lungs and, to a lesser extent, other organs. Transgenic mice expressing Fra-2 experience respiratory distress and fibrosis, accompanied by a significant increase in lung weight and collagen deposition. Histopathological analysis revealed early vascular alterations and increased smooth muscle cell proliferation, followed by interstitial inflammation and collagen deposition [52,53]. The lung pathology mirrored key features of human pulmonary fibrotic diseases, suggesting relevance to human disease mechanisms [47,54,55,56].

Building on our previous study using [^68^Ga]Ga-DATA^5m^.SA.FAPi to image cardiac fibrosis in mouse models for myocardial infarction and transverse aortic constriction [57], we aimed to investigate the potential of this FAPI tracer for imaging pulmonary fibrosis. Specific measurement timepoints were selected corresponding to early, middle, and late disease stages. Since our second mouse model (*fra*-2^tg^) also exhibits fibrotic changes in the heart, liver and thymus, we additionally examined these organs in our study. Our in vivo and ex vivo data were complemented by quantitative immunohistochemistry (IHC) analyses of FAP expression in the lung, as well as histological staining with Sirius Red to visualize pulmonary collagen deposition.

## 2. Results

### 2.1. [^68^Ga]Ga-DATA^5m^.SA.FAPi Uptake in BLM and Control Mice

**Figure 1:**
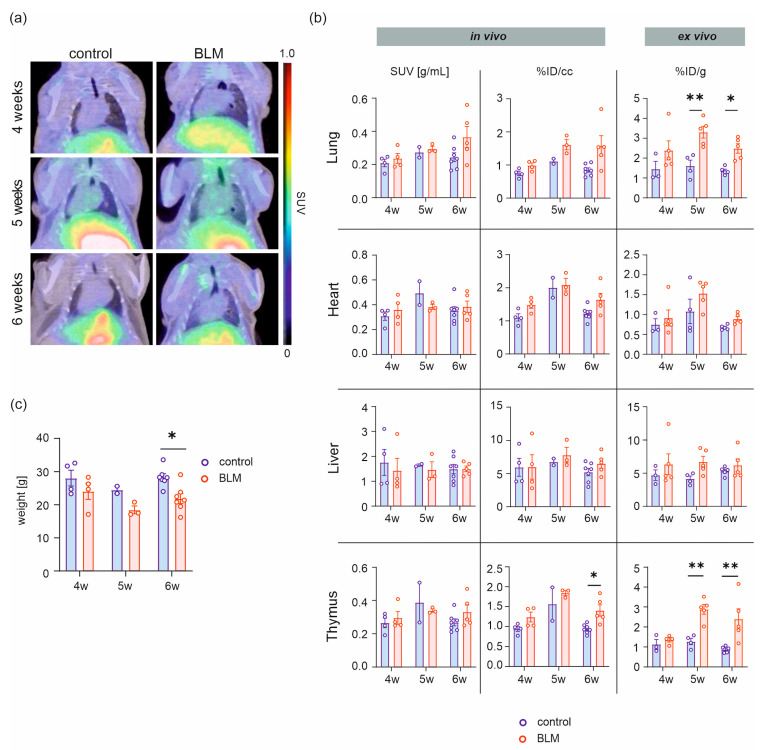
Longitudinal in vivo PET/CT imaging and quantitative analysis of BLM and control mice. Representative [^68^Ga]Ga-DATA^5m^.SA.FAPi PET/CT scans (coronal view) show the uptake of [^68^Ga]Ga-DATA^5m^.SA.FAPi in the lung, heart, liver, and thymus 4, 5, and 6 weeks following the initiation of weekly bleomycin injection (**a**). In vivo and ex vivo analysis of the respective organs (**b**) and quantification of body weight changes during disease progression (**c**). Data are expressed as the mean ± SEM, * *p* > 0.05, ** *p* > 0.01, %ID/cc = percentage injected dose per cubic centimeter, %ID/g = percentage injected dose per gram tissue.

No significant differences in the in vivo uptake of [^68^Ga]Ga-DATA^5m^.SA.FAPi were detected in organs susceptible to systemic fibrosis, including the lung, heart, and liver. However, when focusing on the lung, a trend toward enhanced tracer uptake was consistently observed in the BLM cohorts across all investigated timepoints compared with control mice (Figure 1a,b, Table 1). The body weight normalized in vivo results are presented as standardized uptake values (SUV; g/mL).

In the heart, tracer uptake showed a dynamic pattern: after 4 weeks of treatment, a trend toward higher uptake was observed in the BLM cohort compared with controls (BLM: 0.36 ± 0.05 g/mL, n = 4; control: 0.31 ± 0.03 g/mL, n = 4). This trend reversed at 5 weeks (BLM: 0.38 ± 0.02 g/mL, n = 3; control: 0.49 ± 0.10 g/mL, n = 2) and reverted to the initial pattern at 6 weeks (BLM: 0.39 ± 0.05 g/mL, n = 5; control: 0.35 ± 0.04 g/mL, n = 7) (Figure 1a,b).

In the liver, uptake levels were nearly identical between both groups (4 weeks: BLM: 1.43 ± 0.49 g/mL, n = 4; control: 1.76 ± 0.53 g/mL, n = 4; 5 weeks: BLM: 1.47 ± 0.32 g/mL, n = 3; control: 1.65 ± 0.05 g/mL, n = 2; 6 weeks: BLM: 1.50 ± 0.11 g/mL, n = 5; control: 1.49 ± 0.19 g/mL, n = 7) (Figure 1a,b).

The uptake pattern of the thymus aligned with those of the lung and heart, showing a trend for increased tracer uptake in the BLM cohort compared to the controls at 4 and 6 weeks of treatment (4 weeks: BLM: 0.30 ± 0.04 g/mL, n = 4; control: 0.27 ± 0.03 g/mL, n = 4; 6 weeks: BLM: 0.33 ± 0.04 g/mL, n = 5; control: 0.27 ± 0.02 g/mL, n = 7).

The non-body weight-normalized values (%ID/cc) revealed more distinct differences between the two groups, with only minor disparities in pulmonary uptake and in the thymus at the early stage but increasingly pronounced differences at later timepoints (Figure 1b, Table 1). This trend was also partially reflected in the heart and liver, although the differences between BLM-treated and control mice were less consistent than in the lung. In contrast, a significant difference in the uptake between BLM and the control cohort could be revealed in the thymus at the last measurement timepoint (*p* = 0.01) (Figure 1b).

Ex vivo analysis revealed significant differences between both animal groups at the 5 week (BLM: 3.31 ± 0.29%ID/g, n = 5, control: 1.61 ± 0.29%ID/g, n = 4, *p* = 0.0035) and 6 week timepoints (BLM: 2.49 ± 0.24%ID/g, n = 5; control: 1.36 ± 0.08%ID/g, n = 5, *p* = 0.044) in the lung as well as in the thymus after 5 weeks of treatment (BLM: 2.89 ± 0.24%ID/g, n = 5; control: 1.27 ± 0.16%ID/g, n = 4; *p* = 0.0022). Six weeks post-bleomycin treatment start, the uptake decreased in both cohorts, remaining in significant difference (BLM: 2.40 ± 0.51, n = 5; control: 0.88 ± 0.07%ID/g, n = 5; *p*= 0.0022). No significant differences were observed in heart and liver (Figure 1b).

Analysis of the animal body weight at the first measurement timepoint showed BLM-treated mice weighing 24.10 ± 2.45 g (n = 4) and controls 28.08 ± 2.38 g (n = 4). One week later, weights decreased to 18.47 ± 1.17 g (BLM, n = 3) and 24.5 ± 1.10 g (control, n = 2). At the final measurement after six weeks of treatment, a significant difference in body weight was observed (*p* = 0.013), with BLM mice weighing 21.90 ± 1.51 g (n = 7) and controls 28.30 ± 1.08 g (n = 7) (Figure 1c).

### 2.2. [^68^Ga]Ga-DATA^5m^.SA.FAPi Uptake in fra-2^tg^ Mice and WT Littermates

**Figure 2:**
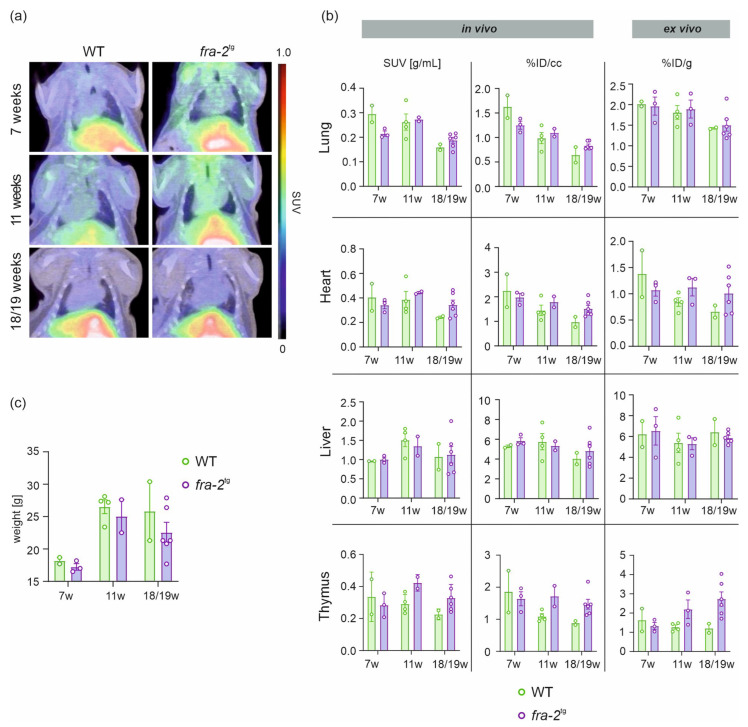
Quantitative analysis of *fra*-2^tg^ and WT mice using longitudinal in vivo PET/CT imaging. Representative [^68^Ga]Ga-DATA^5m^.SA.FAPi PET/CT scans (coronal view) demonstrate uptake of the tracer in the lung, heart, liver, and thymus at 7, 11, and 18/19 weeks of age (**a**). In vivo analysis expressed as SUV (g/mL) and %ID/cc, along with ex vivo analysis expressed as %ID/g (**b**), complemented by body weight changes over time (**c**). Data are expressed as the mean ± SEM, %ID/cc = percentage injected dose per cubic centimeter, %ID/g = percentage injected dose per gram tissue.

In vivo tracer quantification (SUV, %ID/cc) did not reveal significant differences between groups in the lung, heart, liver, or thymus (Figure 2a,b; Table 1). A trend toward lower uptake was observed in the transgenic model at the early stage (7 weeks), whereas at the middle and late stages, *fra*-2^tg^ mice tended to show higher tracer accumulation. Interestingly, pulmonary uptake seemed to decrease with age as it was lower at the final stage (18/19 weeks) compared to earlier timepoints in both cohorts (*fra*-2^tg^ and WT). Ex vivo results confirmed the uptake pattern observed in imaging except for the uptake in the thymus, which seems to increase in the transgenic mice during disease progression (Figure 2b; Table 1).

Analysis of body weight revealed no significant differences between the *fra*-2^tg^ and WT mice. Both cohorts exhibited a marked increase between the early and middle stages (7 weeks: *fra*-2^tg^: 17.23 ± 0.50 g, n = 3; WT: 18.20 ± 0.50 g, n = 2; 11 weeks: *fra*-2^tg^: 25.05 ± 2.55 g, n = 2; WT: 26.53 ± 1.06 g, n = 4). At the late stage, body weight fluctuated considerably in both cohorts (*fra*-2^tg^: 22.57 ± 1.56 g, n = 6; WT: 25.85 ± 4.55 g, n = 2) (Figure 2c).

### 2.3. Histological and Immunohistochemical Analysis of the Lung from BLM, fra-2^tg^, and Control Mice

**Figure 3:**
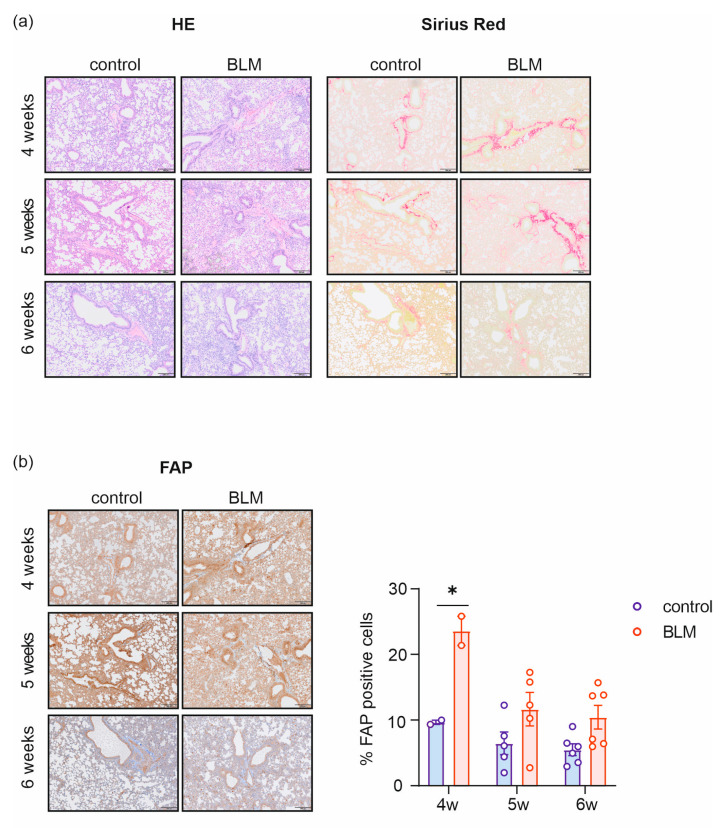
Histology and IHC supplemented with FAP expression analysis of BLM and control lungs. Hematoxylin/Eosin (HE), collagen staining (Sirius Red) (**a**), and FAP staining with corresponding expression analysis (**b**) of representative lungs from BLM and control mice at 4, 5, and 6 week measurement timepoints. The scale bar shown in the right bottom corner corresponds to 200 µm, * *p* > 0.05.

The histological Hematoxylin/Eosin (HE) stainings of BLM-treated mice at 4, 5, and 6 weeks displayed an altered lung architecture compared to their age-matched controls. Sirius Red staining revealed more clearly the enhanced collagen deposition around the vessels (Figure 3a). Perivascular fibrosis was especially evident after 4 and 5 weeks of BLM treatment. Immunohistochemical staining for FAP showed consistently higher expression in BLM-treated mice across all timepoints compared with controls (Figure 3b). Quantitative analysis using StrataQuest software confirmed these findings, with the 4-week BLM-treated group exhibiting significantly higher FAP expression than controls (*p* = 0.0248). Although FAP expression was also elevated in the 5- and 6-week BLM groups, the differences did not reach statistical significance. Overall, the proportion of FAP-positive cells in the BLM group declined over time, decreasing from 23.5% at 4 weeks to 11.6% at 5 weeks and 10.4% at 6 weeks.

**Figure 4:**
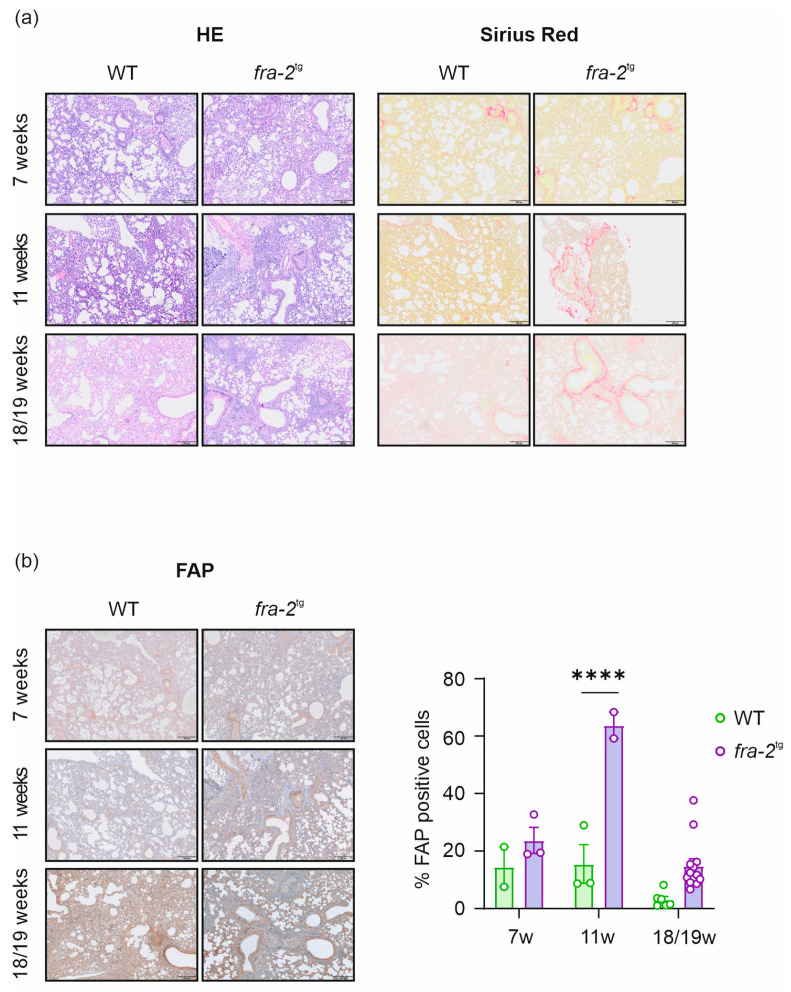
Pulmonary histology and immunohistochemistry (IHC) supplemented with fibroblast activation protein (FAP) expression analysis of *fra*-2^tg^ and WT mice. Hematoxylin/Eosin (HE), collagen staining (Sirius Red) (**a**), and FAP staining with corresponding expression analysis (**b**) of representative lungs from *fra*-2^tg^ and WT mice at 7, 11, and 18/19 weeks of age. Scale bar (bottom right) = 200 µm, **** *p* > 0.0001.

In the *fra*-2^tg^ mice, HE staining revealed restructuring of lung tissue at 7, 11, and 18/19 weeks of age, compared to WT littermates. Collagen accumulation within the lung was less pronounced (Figure 4a) than that observed in the BLM cohort (Figure 3a). Quantitative analysis of FAP expression showed consistently higher levels in *fra*-2^tg^ mice compared with their age-matched WT controls. A statistically significant increase in FAP expression was observed in the 11-week-old *fra*-2^tg^ mice relative to WT controls (*p* < 0.0001), whereas the differences at 7 and 18/19 weeks did not reach statistical significance. The average proportion of FAP-positive cells in *fra*-2^tg^ mice was 23.7% at 7 weeks, which peaked at 63.8% at 11 weeks and declined to 14.8% at 18/19 weeks (Figure 4b).

## 3. Discussion

FAPI tracers emerged as an important tool for molecular imaging of various cancers and are expected to be increasingly useful in the diagnosis of fibrotic diseases such as pulmonary fibrosis. In this pathological process, fibroblasts are activated into myofibroblasts, leading to excessive production of extracellular matrix (ECM) and ultimately resulting in loss of organ function. FAP plays a central role in this pathogenesis, and targeting it with FAPI tracers may provide valuable insights into the causes, progression, and potential therapeutic strategies for fibrotic disease.

A previous study by Sun et al. [11] evaluated the diagnostic accuracy and therapeutic response in BLM intratracheally induced pulmonary fibrosis using [^68^Ga]FAPI-04 PET/CT, demonstrating significantly higher tracer uptake in the BLM cohort compared with controls, beginning in the third week of treatment. Building on this work, our study investigated the potential of [^68^Ga]Ga-DATA^5m^.SA.FAPi for imaging systemic fibrosis in both BLM-induced and transgenic *fra*-2^tg^ mouse models. Therefore, preclinical PET/CT scans were performed in early, middle, and late stages of fibrotic disease corresponding to 4, 5, and 6 weeks of treatment in the BLM-induced mouse model, and 7, 11, and 18/19 weeks of age in the genetically modified model.

Contrary to our hypothesis, in vivo measurements revealed no significant differences in [^68^Ga]Ga-DATA^5m^.SA.FAPi uptake between the fibrotic groups (BLM and *fra*-2^tg^) and their respective controls throughout disease progression. Nevertheless, a trend toward increased pulmonary tracer uptake was observed in the BLM mice, consistent with the findings of Sun et al., although in our study, BLM was administered via peritoneal injection rather than the more commonly used intratracheal route [11,33,35,39,40,41].

This trend was more pronounced in the non-body weight-normalized values (%ID/cc), as the BLM mice exhibited lower body weights compared with controls. [^68^Ga]Ga-DATA^5m^.SA.FAPi uptake in the lung peaked after 5 weeks of treatment, whereas uptake in the controls remained nearly constant across all timepoints. Ex vivo quantification further confirmed a significantly higher tracer uptake in the BLM cohort at both 5 and 6 weeks compared with controls. In contrast, IHC analysis revealed the highest pulmonary FAP expression at 4 weeks, which gradually declined at later timepoints. This phenomenon, also reported in previous studies [5,11], may reflect characteristics of this specific mouse model, which initially simulates an early inflammatory phase of idiopathic pulmonary fibrosis (IPF) before transitioning into a fibrotic response. It may also indicate that FAP plays a more prominent role during the acute fibrotic phase, dominated by activated fibroblasts, than during scar maturation.

We included the *fra*-2^tg^ mouse model to investigate systemic fibrosis associated with perivascular changes and systemic inflammation [52]. The lungs of these mice exhibit histopathological features similar to those found in human non-specific interstitial pneumonia (NSIP) and IPF-related usual interstitial pneumonia, including fibrotic remodeling [54,55]. Similarly to the BLM model, in *fra*-2^tg^ mice, FAP expression was highest not at the late stage of disease but already at the onset (11 weeks). This is consistent with the findings of Eferl et al. [47], who reported that histological differences between transgenic and WT mice become evident towards the middle stage of the disease. Notably, the obliteration of pulmonary arteries, along with perivascular inflammatory infiltrates, was observed at 12 weeks of age, preceding the onset of fibrosis by approximately two weeks [54]. Based on these findings, we aimed to determine the earliest timepoint at which the FAPI tracer could effectively detect pathological changes. No significant differences in [^68^Ga]Ga-DATA^5m^.SA.FAPi uptake were observed when comparing *fra*-2^tg^ and WT mice in either in vivo or ex vivo quantifications of all included organs. The unchanged pulmonary tracer uptake may be attributed to vascular obliteration, resulting in localized impairment of tracer distribution. However, in vivo imaging highlighted increased [^68^Ga]Ga-DATA^5m^.SA.FAPi uptake in the thymus (Figure 2a), an organ known to undergo fibrotic changes in this animal model, as described by Eferl et al. [47]. Consistently, we observed a trend toward increased tracer uptake in the thymus of transgenic mice during the middle and late stages of disease (Figure 2b). A similar pattern was observed in the BLM model, where tracer uptake increased significantly after 5 and 6 weeks of treatment (Figure 1a,b).

The body weight itself (Figure 2c) reflected aging and disease progression, showing an increase from the first (7 weeks) to the second timepoint (11 weeks) of measurement, where a difference between *fra*-2^tg^ and WT mice also emerged. In contrast to the weight of WT animals, which seemed to remain stable, the body weight of the transgenic mice declined in view of disease severity.

Although analysis of pulmonary FAP expression confirmed the published findings [5,11,13,47,48,54,55] and revealed a significant increase at the early to middle stages of the disease (Figure 3a,b and Figure 4a,b), this pattern was not consistently mirrored by in vivo or ex vivo tracer measurements. Instead, [^68^Ga]Ga-DATA^5m^.SA.FAPi uptake tended to be higher at later stages. These discrepancies may also reflect limitations inherent to our study design. The varying and sometimes low sample size was due to occasional technical issues during scanning, the short half-life of gallium-68, and the limited availability of the precursor. To maximize the efficient use of each [^68^Ga]Ga-DATA^5m^.SA.FAPi batch, additional animals were injected solely for biodistribution analysis, in parallel with those that underwent PET/CT imaging. The small differences in tracer uptake between fibrotic animals and their respective controls—particularly in the *fra*-2^tg^ model—may additionally be attributed to the high background signal of this FAPI tracer or progressive vascular occlusion. In the BLM mouse model, the high background, combined with the use of intraperitoneal rather than intratracheal BLM administration (to better align with the transgenic model), may have contributed to the small differences in tracer uptake between the control and disease cohorts. Alternative FAPI tracers could potentially provide greater contrast and reveal significant group differences, making them more advantageous for molecular imaging. It is also important to note that variability observed in our second animal model may be partly attributed to differences in sex. As reported in the literature, females are more affected by autoimmune diseases, with women representing over 80% of patients diagnosed with primary Sjögren’s syndrome, systemic lupus erythematosus, primary biliary cirrhosis, autoimmune thyroid disease, and systemic sclerosis [13,58]—conditions that correlate with the *fra*-2^tg^ mouse model.

Although our in vivo results did not show significant differences at all measurement timepoints, the data revealed a trend suggesting that FAP inhibitor tracers could be valuable for imaging pulmonary fibrotic diseases. In conclusion, our findings align with previous studies indicating that FAPI tracers generally hold potential for fibrosis imaging, although [^68^Ga]Ga-DATA^5m^.SA.FAPi may not be the most suitable tracer, as also evident in our prior study on cardiac fibrosis imaging [57].

## 4. Materials and Methods

### 4.1. Radiosynthesis of [^68^Ga]Ga-DATA^5m^.SA.FAPi

Unless otherwise specified (Table 2), the chemicals used in this study were obtained from Sigma Aldrich (St. Louis, MO, USA). The precursor preparation followed the method described by Moon et al. [59], while detailed procedures for the manual radiosynthesis are provided by Weissenböck et al. [57]. In total, 38 successful syntheses were achieved across this and the previous study [57]. Quality control by high performance liquid chromatography (HPLC) revealed a radiochemical purity of 98.5 ± 1.4%. Thin layer chromatography (TLC) analysis resulted in Rf values of 0.1–0.2 for the product and 0.7–0.9 for free radio metal in citrate buffer, and Rf values of 0.55–0.75 for the product and 0.02–0.04 for colloid in NH_4_OAc buffer.

### 4.2. Mouse Models

#### 4.2.1. Bleomycin-Treated Mice

Male C57BL/6 mice (10–12 weeks old; Charles River Laboratories, Sulzfeld, Germany) were housed in a specific pathogen-free facility at 23 °C, a 12:12 h day/night mode, and with food and water ad libitum. The animals were briefly anesthetized (2% isoflurane in oxygen) and administered 100 µL (3 Units/mL) bleomycin sulfate (B5507, Sigma Aldrich, St. Louis, MO, USA) intraperitoneally (i.p.). The compound was dissolved in physiological saline solution. This treatment was performed twice a week over a period of 4, 5, and 6 weeks, respectively, to induce systemic fibrosis. Control mice were injected with physiological saline solution under the same conditions over the same period. Both cohorts were housed in different cages.

#### 4.2.2. *fra*-*2^tg^*/WT Mice

Female and male mice were bred, maintained on a C57BL/6 background, and housed in a specific pathogen-free facility as described for the BLM model. The *fra*-2^tg^ (*H2kb-fosl2-LTR*, MGI:3813493) model was described by Eferl et al. [47,54]. Cohorts of *fra*-2*^tg^* and their wildtype (WT) littermates aged 7 to 19 weeks were used for the experiments.

### 4.3. In Vivo Imaging and Data Analysis

To investigate the progression of fibrosis in multiple organs, mice were studied during 4, 5, and 6 weeks of bleomycin injection. Naïve mice served as the control group. Genetically modified *fra*-2^tg^ and corresponding WT mice were examined at ages of 7, 11, and 18/19 weeks. Anesthetized (2–3% isoflurane in oxygen) mice were injected intravenously (i.v.) into the lateral tail vein with 15–20 MBq of [^68^Ga]Ga-DATA^5m^.SA.FAPi in a volume of 100 to 150 µL. Following a 45 min distribution time, a static 10 min PET and 5 min CT scan, using a Inveon^®^ Multi-Modality microPET/CT Scanner (Siemens Healthineers, Erlangen, Germany), was performed. The mice were maintained under anesthesia on heated beds and their respiratory rate was monitored from the time of tracer injection until sacrifice.

CT raw data were reconstructed using an OSEM algorithm with integrated standard mouse beam-hardening correction and noise reduction, employing a 1024 × 1024 matrix and a 97.56 µm pixel size. The calibrated CT image data were converted to Hounsfield Units (HU). PET data were organized into three-dimensional sinograms and reconstructed with a Feldkamp algorithm, omitting scatter correction, but applying a ramp filter (256 × 256 matrix). The data were normalized and corrected for random events, dead time, and radioactivity decay. A calibration factor was used to convert the activity data into absolute concentration units.

For further imaging analysis, processing, and quantification of the static microPET/CT scans, the Inveon Research Workplace (IRW) software version 4.2 (Siemens Healthineers, Erlangen, Germany) was consulted. The CT images were co-registered with the corresponding PET scans and regions of interest (ROIs) were delineated to create volumes of interest (VOIs). The respective tracer uptake was normalized to the injected dose and the animal’s weight. To facilitate comparison of the results across the animals, data were presented as standardized uptake values (SUV; g/mL). The whole imaging and postprocessing workflow are also described in Weissenböck et al. [57].

### 4.4. Ex Vivo Biodistribution

Following the PET/CT scan, anesthetized mice were euthanized by cervical dislocation. Organs (lung, heart, liver, and thymus) were harvested for radioactivity measurement using a gamma counter (2480 Automatic Gamma Counter Wizard^2^ 3”, Perkin Elmer, Waltham, MA, USA). For the biodistribution analysis, gamma counter data were decay-corrected and results were expressed as the percentage of the injected dose of radioactivity per gram of tissue (%ID/g).

### 4.5. Histological and Immunohistochemical Analysis

HE, Sirius Red, and IHC were performed using standard protocols on FFPE consecutive sections. For IHC, the following antibody was used: FAP (PA5-99313, 1:100 dilution, Invitrogen, Carlsbad, CA; USA). The specific signal was developed under microscopic control. Quantitative analyses were performed with StrataQuest software version 8.0.61 (TissueGnostics GmbH, Vienna, Austria).

### 4.6. Statistical Analysis

Statistical analyses were performed in GraphPad Prism 8.2.1 and 9.2.0 (GraphPad Software, Inc., San Diego, CA, USA). Values are represented as arithmetic means ± standard error mean (SEM). Statistical significance was calculated with two-way ANOVA test. Significance is indicated in the figures by *: *p*-value < 0.05; **: *p*-value < 0.01; ***: *p*-value < 0.001; ****: *p*-value < 0.0001.

## 5. Conclusions

Our findings complement and extend previous observations, supporting the potential of FAPI tracers as molecular imaging agents for pulmonary fibrosis. Although no significant differences were observed at all timepoints, the data revealed a consistent trend suggesting that FAP inhibitor tracers may be valuable for imaging fibrotic lung disease.

## Figures and Tables

**Table 1:** Overview of all pulmonary in vivo and ex vivo data at all measurement timepoints (*: *p*-value < 0.05; **: *p*-value < 0.01).

Timepoint	Mouse Model	*In Vivo*			*Ex Vivo*	
		%ID/cc	SUV [g/mL]	*n*	%ID/g	*n*
**4 weeks**	Control	0.75 ± 0.07	0.21 ± 0.02	4	1.45 ± 0.39	3
	BLM	0.99 ± 0.08	0.24 ± 0.03	4	2.39 ± 0.48	5
**5 weeks**	Control	1.11 ± 0.08	0.27 ± 0.03	2	1.61 ± 0.29	4
	BLM	1.62 ± 0.16	0.30 ± 0.01	3	3.31 ± 0.29 **	5
**6 weeks**	Control	0.85 ± 0.07	0.24 ± 0.03	7	1.36 ± 0.09	5
	BLM	1.58 ± 0.31	0.37 ± 0.06	5	2.49 ± 0.24 *	5
**7 weeks**	WT	1.63 ± 0.23	0.30 ± 0.03	2	2.02 ± 0.06	2
	*fra*-2^tg^	1.25 ± 0.08	0.22 ± 0.01	3	1.97 ± 0.22	3
**11 weeks**	WT	0.99 ± 0.11	0.26 ± 0.03	4	1.81 ± 0.17	4
	*fra*-2^tg^	1.10 ± 0.08	0.27 ± 0.01	2	1.89 ± 0.22	3
**18/19 weeks**	WT	0.65 ± 0.16	0.16 ± 0.01	2	1.43 ± 0.02	2
	*fra*-2^tg^	0.84 ± 0.03	0.19 ± 0.01	6	1.50 ± 0.15	6

**Table 2:** Materials used in this study.

Product	Product Number	Company
DATA^5m^.SA.FAPi Precursor		Johannes Gutenberg University Mainz, Mainz, Germany
DMF	047390.AE	Thermo Fisher Scientific, Waltham, MA, USA
0.9% NaCl	3505731	Braun, Melsungen, Germany
HCl Reag.Ph.Eur. 0.1N	6789	Carl Roth GmbH, Karlsruhe, Germany
Cartridge SEP-PAK C18 light	WAT023501	Waters Corporation, Milford, MA, USA
iTLC-SG plates	SGI0001	Agilent Technologies, Santa Clara, CA, USA
^68^Ge/^68^Ga Generator	438495	IRE Galli Eo^®^, Fleurus, Belgium
Dry block heater	0004025100	IKA^®^, Staufen, Germany
HPLC VWR Hitachi Chromaster	5410 UV Detector, 5310 Column Oven, 5160 Pump	VWR, Tokyo, Japan
Chromolith^®^ Performance RP-18e (100–4.6 mm) column	102129	Merck KGaA, Darmstadt, Germany
TLC scanner Elysia Raytest GITA*	B00002881	Elysia-Raytest, Straubenhardt, Germany
CLARITY 7.3 software	C50	Data Apex, Prague, Czech Republic
Gina Star TLC 6.3		Elysia-Raytest, Straubenhardt, Germany

## Data Availability

The original contributions presented in this study are included in the article. Further inquiries can be directed to the corresponding author.

## Abbreviations

The following abbreviations are used in this manuscript:

AL	Amyloid light chain
BLM	Bleomycin
CT	Computed tomography
DATA	2,2′-(6-((carboxymethyl)amino)-1,4-diazepane-1,4-diyl) diacetic acid
FAP	Fibroblast activation protein
FAPI	FAP inhibitor
*fra*-2	Fos-related antigen 2
Ga	Gallium
HCl	Hydrochloric acid
HE	Hematoxylin/Eosin
HPLC	High-performance liquid chromatography
HU	Hounsfield Units
ID	Injected dose
IHC	Immunohistochemistry
IPF	Idiopathic pulmonary fibrosis
i.p.	Intraperitoneal
i.v.	Intravenous
MeCN	Acetonitrile
NSIP	Non-specific interstitial pneumonia
NH_4_OAc	Ammonium acetate
PET	Positron emission tomography
SA	Squaric acid
SUV	Standardized uptake value
Tg	Transgenic
TLC	Thin layer chromatography
WT	Wildtype
